# Impact of Particle Size of Ceramic Granule Blends on Mechanical Strength and Porosity of 3D Printed Scaffolds

**DOI:** 10.3390/ma8084720

**Published:** 2015-07-24

**Authors:** Sebastian Spath, Philipp Drescher, Hermann Seitz

**Affiliations:** Faculty of Mechanical Engineering and Marine Technology, University of Rostock, Justus-von-Liebig Weg 6, 18059 Rostock, Germany; E-Mails: sebastian.spath@outlook.com (S.S.); philipp.drescher@uni-rostock.de (P.D.)

**Keywords:** 3D printing, scaffold, particle size, porosity, mechanical strength

## Abstract

3D printing is a promising method for the fabrication of scaffolds in the field of bone tissue engineering. To date, the mechanical strength of 3D printed ceramic scaffolds is not sufficient for a variety of applications in the reconstructive surgery. Mechanical strength is directly in relation with the porosity of the 3D printed scaffolds. The porosity is directly influenced by particle size and particle-size distribution of the raw material. To investigate this impact, a hydroxyapatite granule blend with a wide particle size distribution was fractioned by sieving. The specific fractions and bimodal mixtures of the sieved granule blend were used to 3D print specimens. It has been shown that an optimized arrangement of fractions with large and small particles can provide 3D printed specimens with good mechanical strength due to a higher packing density. An increase of mechanical strength can possibly expand the application area of 3D printed hydroxyapatite scaffolds.

## 1. Introduction

The use of 3D printing for the fabrication of scaffolds for bone tissue engineering is very promising [1]. It is possible to design custom implants with the aid of patient CT data. The non-existing design limitations and harmless use of synthetic materials could make 3D printing very valuable in reconstructive surgery [2,3,4].

Anatomical information obtained from patients can be used to design and optimize the implant for a bone defect. 3D printing allows manufacturing matrices with complex shapes as well as designed internal channel network to mimic bone structures. Today, different synthetic bone replacement materials are available. These materials are mostly from the group of Calciumphosphates. Especially β-Tricalciumphosphate (β-TCP) and Hydroxyapatite (HA) are favored bulk materials for synthetic bone grafts. These are materials with enormous potential as a bone substitute because of its similarity to the inorganic components of human bone [5,6].

Synthetic HA granule-blends can be processed in a 3D printer [7] and post-processed in a sintering furnace to improve the mechanical properties. However, by sintering the degradation behavior of the material can be influenced [8]. For successful 3D printing a smooth and homogeneous powder bed surface is necessary. To achieve this, the granular raw material must feature a good flowability. Flowability of raw HA is not sufficient for plane powder recoating but can be increased by using spherical granule blends, which are fabricated by spray drying from calcium phosphate slurries [7].

The spray-dried granule blends lead—due to their nearly spherical shape—to a high microporosity of 3D printed parts. This property is desirable for good cell attachment, differentiation [9], proliferation [10] and spreading [11]. The colonization and proliferation of osteoblasts have been investigated with HA and have shown positive results compared to commonly used bone replacement materials [6]. Above that, micro-porosity (diameter < 10 μm) allows body fluid circulation [12]. A higher microporosity provides an increase in rate and volume of bone ingrowth [13], bone contact [14] and resorption [9]. However, inadequate mechanical strength prevents the use of 3D printed parts in many applications in reconstructive surgery.

The porosity is directly influenced by particle size and particle-size distribution and therefore in relation to the mechanical strength of the part. The possibility of increasing the mechanical strength (or decreasing porosity) by using a specific particle size or an optimized combination of different particle sizes is well known from other technical fields [15,16]. It is possible to increase the packing density with bimodal packing through a specific combination of large and small particles [17,18]. This can be explained by the fact that the small particles fill void spaces between the large particles without dilating the overall system volume [19].

The underlying 3D printing process of this study and several processing aspects has been published previously [7,20,21]. Especially, the influence of particle sizes on bulk and tapped density, which has significant effects on the condition of the powder bed during printing, was investigated in a former study [22]. Various surveys also investigated the mechanical properties, the biocompatibility as well as the cell behavior of various cells seeded on 3D printed HA and TCP scaffolds [6,23,24,25,26,27,28,29,30,31]. Further approaches focus on the infiltration of 3D printed TCP scaffolds with biodegradable polymers and biomolecules for local drug delivery [32] and on the application of BMP-2 onto 3D printed scaffolds for heterotopic bone induction [33,34], as well as the feasibility of using scaffold-based tissue engineering in alveolar cleft osteoplasty [35].

The possibility of influencing the mechanical strength of 3D printed implants by varying particle sizes and particle-size distributions is an important field of research [36]. By increasing the mechanical strength, such scaffolds could also be used in load-bearing regions. The aim of this study is to determine the impact of granule blends with different particle sizes and different particle size distributions as well as mixtures (combination of different particle sizes) on mechanical strength and porosity of 3D printed parts.

## 2. Materials and Methods

### 2.1. 3D Printing

3D printing builds physical 3D models directly from computer data [37]. During the process, unbound powder is wetted with binder successively [21]. At first a thin layer of powder is coated on the building platform. Afterwards—according to computer data—a liquid binder is selectively printed on the layer and bonds the wetted regions. After the completion of a layer, the building box moves down by the value of the layer thickness and a new layer of powder is deposited onto the previous. Repeating the described steps creates three-dimensional parts which can be removed after a certain drying time from the loose and unbound powder.

The used 3D printer is a non-commercial system with a build envelope of 100 mm × 100 mm × 100 mm (*x*, *y*, *z*). The print head functions as a micro-mechanical valve. The printing binder is an aqueous solution of dextrin (20 wt %) and saccharose (2.5 wt %). The printing resolution is 0.25 mm in all axes.

The fabricated 3D-printed ceramic green bodies were consolidated at a temperature of 1250 °C in a high temperature furnace (HTC 08/14, Nabertherm, Germany) in ambient air. During this post processing treatment the organic binder undergoes pyrolysis and the 3D printed specimens obtain their final properties. A previous study has shown that the sintering shrinkage for this material was typically in the range of 30%. This was taken into consideration by scaling the data set accordingly [20].

More details on the fabrication and sintering process of the 3D printed specimens can be comprehended in a previous study [21].

### 2.2. Granule Blends

A spray-dried hydroxyapatite granule blend was used (HA SP19, BioCer Entwicklungs-GmbH, Bayreuth, Germany) [7]. The granule blend was prepared and analyzed (see Table 1) according to a previous study [22].

**Table 1:** Fractions of hydroxyapatite granule blend.

Fraction	Notation
<32 μm	<32
32–45 μm	32–45
45–63 μm	45–63
63–80 μm	63–80
80–100 μm	80–100
100–125 μm	100–125
>125 μm	>125
entire granule blend	EG

The entire spray-dried granule blend (EG) was sieved for 10 min with amplitude of 1.5 mm using a sieve shaker (Retsch A200 Digit, Retsch GmbH, Haan, Germany) and thereby separated into the following particle-size fractions.

In addition, three bimodal fraction mixtures, as shown in Table 2, were prepared and tested. These fraction mixtures were made by adding 15 wt %, 25 wt % and 35 wt % of fraction 32–45 to the fraction >125 respectively.

**Table 2:** Bimodal mixtures.

Fraction	Notation
>125 μm + 15 wt % 32–45 μm	>125, 15%
>125 μm + 25 wt % 32–45 μm	>125, 25%
>125 μm + 35 wt % 32–45 μm	>125, 35%

In order to specify the actual particle size distribution (PSD) of the fractions and mixtures after sieving, particle size distribution analysis was performed by laser granulometry (1064, Cilas, Orleans, France). The values for D_10_, D_50_ and D_90_ were determined. D_50_ is defined as the diameter where half of the particles lie below this value. Accordingly, 90% of the distribution lies below the D_90_, and 10% lies below the D_10_.

Optical examination of the granule blend was performed by scanning electron microscopy (SEM) analysis (XL30, Philips, Amsterdam, The Netherlands).

### 2.3. Specimens

Several specimens were designed with SolidWorks (Dassault Systèmes SolidWorks Corp., Concord, MA, USA) CAD software. After exporting the virtual model as a STL file, it is sliced into a stack of two-dimensional bitmap-files with the software Rapix3D (FORWISS Passau, Germany), which represent the corresponding cross-sections of the part to be built.

In accordance to the international standard DIN EN ISO 604, cylindrical specimens with a diameter of 10 mm and a height of 14 mm were designed for mechanical testing. Another cylindrical specimen with a diameter of 7.5 mm and a height of 5 mm was designed for porosity analysis. Five specimens for analysis of mechanical strength and porosity were manufactured for each fraction and mixture, respectively.

The mechanical strength was tested using a uniaxial testing system Zwicki (Zwick GmbH & Co. KG, Ulm, Germany) with a 5 KN load cell. Determination of porosity was performed by Archimedes principle [38,39,40] in accordance to the international standard DIN EN 623-2 using the LAUDA tensiometer TD 1 C (LAUDA DR. R. Wobser GmbH & CO. KG, Lauda-Koenigshofen, Germany). At least three measurements were performed.

The mass was measured with a SBC31 (Scaltec Instruments GmbH, Heiligenstadt, Germany) precision scale. 3D printed parts were analyzed by SEM (XL30, Philips, Amsterdam, The Netherlands).

## 3. Results and Discussion

The spray dried granule blends were successfully fractionated via sieving and three additional bimodal mixtures of fractions with small and large diameters were prepared. The fractionated granule-blends, mixtures as well as the entire granule-blends were characterized optically via laser granulometry.

### 3.1. Particle Size Distribution

The results of the particle size distribution are shown in the following Table 3. It can be seen that the respective fractions do not perfectly match the nominal particle size distribution.

**Table 3:** Results from analysis of particle size distribution (PSD).

Fractions	D_10_ [μm]	D_50_ [μm]	D_90_ [μm]
<32	8.8	20.3	31.0
32–45	11.4	28.3	43.7
45–63	15.1	40.2	59.2
63–80	45.0	63.8	88.3
80–100	60.0	80.2	108.4
100–125	72.4	97.7	132.8
>125	82.9	125.5	178.8
EG	22.5	58.8	107.4
>125, 15%	40.2	122.3	173.2
>125, 25%	36.9	119.9	174.6
>125, 35%	34.4	118.7	175.1

**Figure 1:**
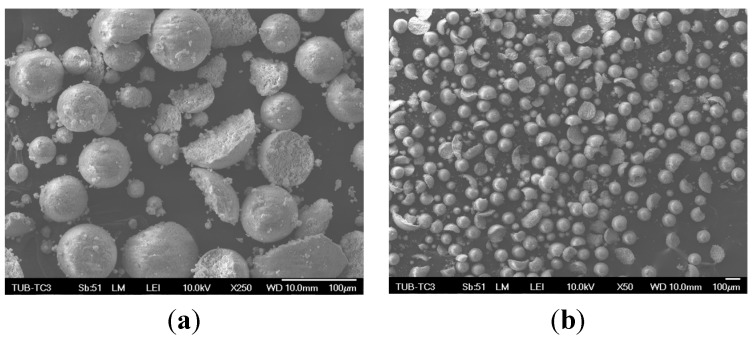
SEM image of broken hydroxyapatite granule-blends with 250× (**a**) and 50× (**b**) magnification.

The entire granule blend (EG) has the largest range of particle sizes. Beyond that a relative large range of particle sizes is observable for the three smallest fractions <32, 32–45 and 45–63, respectively. Despite extensive sieving, fine particles cannot be sieved out entirely and remain in larger fractions. D_90_ of some fractions (63–80, 80–100, 100–125) suggests that there are particles or agglomerates with a larger diameter than the mesh size of the corresponding sieve would theoretically allow [41]. As seen on the SEM images (Figure 1) an explanation is that there are fragments of granule blends, which fit through the meshes of the respective sieves. During the particle size analysis these broken particles were assigned—due to their rudimentary spherical shape—as a larger diameter (possibly original diameter before breaking) by the computational algorithm of the laser granulometer.

### 3.2. Optical Analysis

SEM images were taken to analyze the quality of the particles. Figure 1 shows that the morphology of the particles is not always fully spherical.

**Figure 2:**
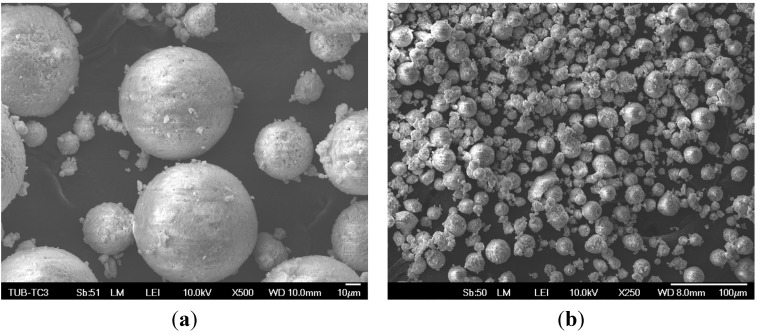
Spherical particles in entire granule-blends (EG) (**a**) and with irregularly shaped particles in fraction <32 (**b**).

Figure 2 shows the entire granule blend and demonstrates the mainly spherical shape of the particles. Beyond that very fine particles adhere on the bigger particles and show their tendency for agglomeration with decreasing diameter. Some broken particles are observable. It can be stated that with decreasing diameter, granule blend shapes will become more irregular.

**Figure 3:**
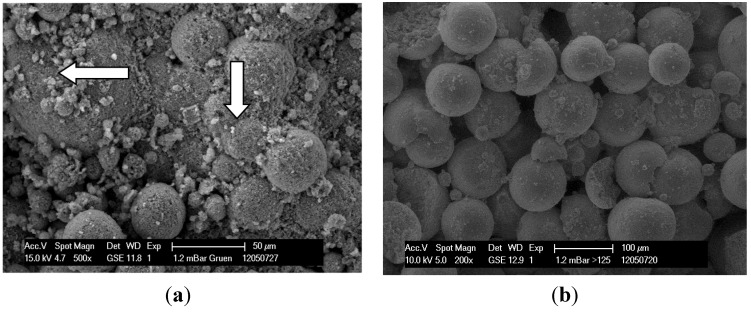
Green part with residual binder (**a**) and a sintered 3D printed part (**b**).

Figure 3a shows the surface of a 3D printed green part. The green part is the part before post-processing. In this case the sintering process, which burns out the organic components and fuses the ceramic particles together. Organic components of the binder (see arrows) can be seen, which bind the particles together and lead to an initial strength necessary for post processing. Figure 3b shows the sintered 3D part. The organic components are burned out completely. Observable is the spherical shape of the particles which causes a high microporosity, depending on particle size.

### 3.3. Mechanical Strength

**Figure 4:**
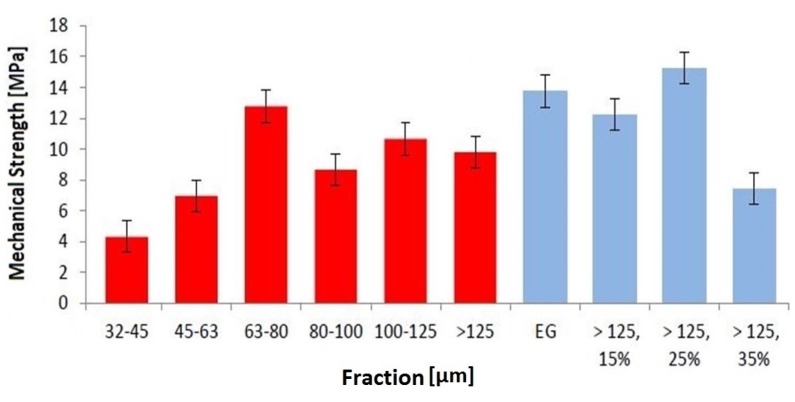
Mechanical strength of 3D printed parts.

Test specimens were successfully printed, sintered and analyzed. Figure 4 shows results from the measurement of the mechanical strength in relation of the fraction used and thus the influence of particle size. Five specimens were prepared for each fraction and each bimodal mixture. Mechanical results show significant dependencies on the used fraction, and thus to their particle size. Unfortunately, the fraction <32 did not deliver enough material to build samples for compressive strength analysis.

Evaluation of the mechanical properties shows that 3D printed test specimens, which were manufactured by using EG, have a higher compressive strength (13.7 MPa) than single fractions. In conclusion, the wide PSD of the entire granule blend lead to a higher mechanical strength with a low porosity of the 3D printed parts, compared to a narrow PSD. It can also be assumed that with decreasing particle sizes the mechanical strength increases as well, except the fraction 63–80. Surprisingly, this fraction has a similarly high mechanical strength as EG. This might be due to the fact that the fractions do not perfectly match the nominal PSD. The fraction 63–80 could therefore consist of particles that are favorable for packing density and therefore the mechanical strength. The morphology of the particles could also have a significant impact on the porosity and therefore on the mechanical results since the particles cannot be considered completely spherical.

According to previous studies, the mechanical strength can be increased by the addition of fine particles to a fraction with larger particle sizes [18]. In comparison to the mechanical strength of fraction >125, an addition of 15 wt % of the fraction 32–45 causes an increase of almost 25%. With an addition of 25 wt %, the mechanical strength exceeds the strength of fraction >125 by 55% and even by 11% of entire granule blend. A higher content of fine particles (>125, 35%), however, reduces mechanical strength, as seen in Figure 4.

### 3.4. Porosity

**Figure 5:**
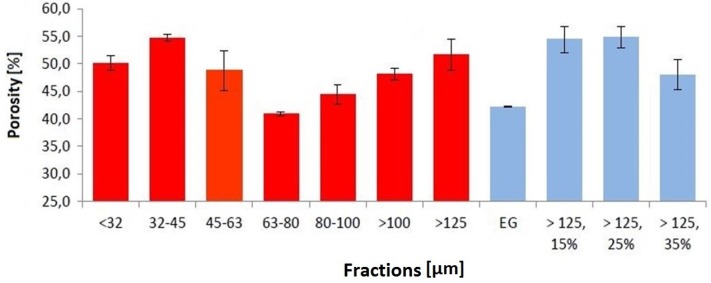
Porosity of 3D printed parts.

The porosity of sintered 3D printed parts was investigated using the Archimedes principle. Figure 5 shows the results from the porosity measurements. The diagram also shows that a dependency of particle size and porosity could not be detected. This might be due to the fact that all fractions still contain particles that do not belong to the fraction and therefore distort the results. Considering an ideal fraction, however, a correlation can be expected.

Specimens manufactured from fraction 63–80 and EG show to have the lowest porosity of all tested granule blends. The fractions with the smallest particles lead to specimens with very high porosities. However, a specimen manufactured with a bimodal distribution can have a higher mechanical strength than specimens with a wide particle size distribution like the EG [42]. This is in accordance to the theory of bimodal granule blends that shows that a specific ratio of particle sizes can lead to the highest packing density [43]. On the other hand, the bimodal granule blends with this material show a higher porosity which is contrary to the theory. This can be explained by the fact that the mixture of the two fractions, are not ideally adjusted for a high packing density.

**Figure 6:**
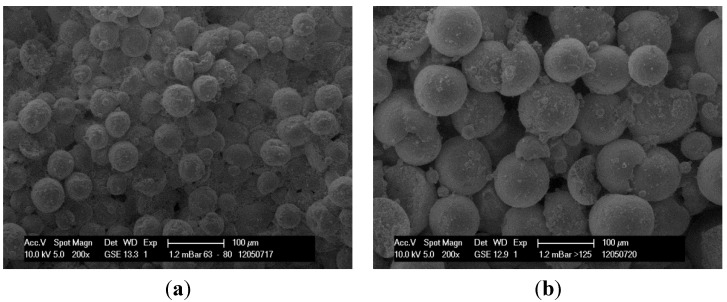
SEM image of specimen made of fraction 63–80 (**a**), SEM image of specimen made of fraction >125 (**b**).

Surface composition and porosity of specimens can be illustrated by SEM images. Specimens made from the fraction 63–80 (Figure 6a) have a dense surface resulting in low porosity. In contrast, fraction > 125 (Figure 6b) shows a high porous structure of the specimen surface.

The following SEM images show differences in the surface condition of specimens made of bimodal granule blend (>125, 25%) and granule blends with wide particle size distribution (EG). Optical investigation of the surface confirms a higher porosity of the bimodal mixture in comparison to the EG due to a more effective filling of the voids.

**Figure 7:**
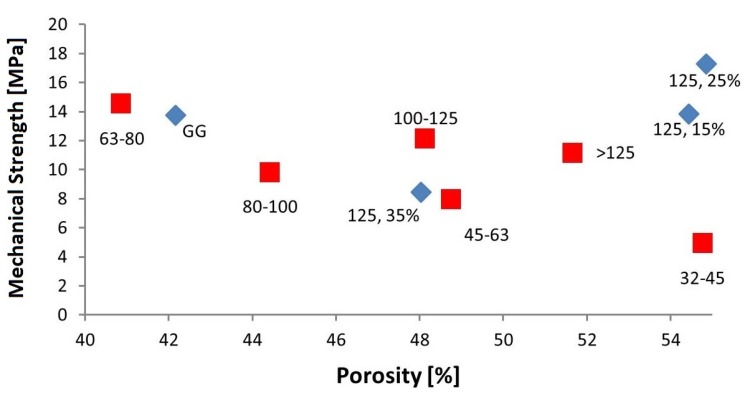
Mechanical strength and porosity of 3D-printed specimen.

It can be seen in Figure 7 that there is a weak correlation between mechanical strength and porosity. The diagram also shows that a dependency of particle size and porosity could not be detected. In general, the smaller the unimodal particle size the higher the porosity with uniform particle morphology due to adhesive forces. Extensive research has been carried out in this field [44,45,46,47]. However, the results differ from the expected findings from theoretically packing density because the morphology of the particles of the investigated granule blends more or less deviate from the spherical shape. Another reason is that the fractions still contain particles that do not belong to the specific fraction and therefore distort the results.

**Figure 8:**
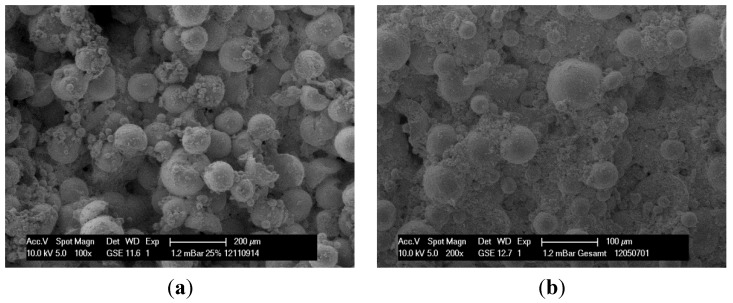
Specimen made of bimodal mixture >125, 25% (**a**), Specimen made of entire granule-blends (EG) with a wide particle size distribution (**b**).

However, there is a clear improvement of mechanical strength of the bimodal fractions. Samples made from the bimodal granule blend (>125, 25%, as seen in Figure 8a) can have—despite of a higher porosity—higher mechanical strength than samples made of granule blends with a wide particle size distribution like the EG. This is to be expected since an optimized packing of spherical particles should lead to higher mechanical strength [42].

## 4. Conclusions

It is possible to increase the packing density of granule blends with an addition of fine particles to larger granule-blends [22]. Therefore, the increase of mechanical strength of the 3D printed specimens was expected. By adding 25 wt % of fine particles (fraction 32–45) to the fraction >125 the mechanical strength was increased by 55% compared to the strength of the single fraction >125. In this study, it was shown that by adding more than 25 wt % of the fine particles reduces the mechanical strength of the sintered specimens. However, the porosity of sintered specimens manufactured with these combined granule blends does not decrease. A direct correlation between particle size diameter and porosity after sintering could not be detected. This might be the cause of the sieving process which leads to higher particle size distributions than the ideal fraction and imperfect spherical particles. This means that not only is the density of raw materials crucial for the condition of sintered specimens but a different sintering behavior of granule-blends with different particle sizes and the combination of them are also important.
